# NADP^+^ Binding to the Regulatory Subunit of Methionine Adenosyltransferase II Increases Intersubunit Binding Affinity in the Hetero-Trimer

**DOI:** 10.1371/journal.pone.0050329

**Published:** 2012-11-26

**Authors:** Beatriz González, Francisco Garrido, Rebeca Ortega, Marta Martínez-Júlvez, Ainhoa Revilla-Guarinos, Yolanda Pérez-Pertejo, Adrián Velázquez-Campoy, Julia Sanz-Aparicio, María A. Pajares

**Affiliations:** 1 Departamento de Cristalografía y Biología Estructural, Instituto de Química-Física “Rocasolano” (CSIC), Madrid, Spain; 2 Instituto de Investigaciones Biomédicas “Alberto Sols” (CSIC-UAM), Madrid, Spain; 3 Departmento de Bioquímica y Biología Molecular y Celular, Universidad de Zaragoza, Zaragoza, Spain; 4 Instituto de Biocomputación y Física de Complejos, Unidad Asociada IQFR-BIFI, Mariano Esquillor s/n, Edificio I+D, Campus Rio Ebro, Zaragoza, Spain; 5 Departamento de Farmacología y Toxicología (INTOXCAL), Universidad de León, Campus de Vegazana s/n, León, Spain; 6 Fundacion ARAID, Diputación General de Aragón, Zaragoza, Spain; 7 Molecular Hepatology Group, IdiPAZ, Madrid, Spain; Indian Institute of Science, India

## Abstract

Mammalian methionine adenosyltransferase II (MAT II) is the only hetero-oligomer in this family of enzymes that synthesize S-adenosylmethionine using methionine and ATP as substrates. Binding of regulatory β subunits and catalytic α2 dimers is known to increase the affinity for methionine, although scarce additional information about this interaction is available. This work reports the use of recombinant α2 and β subunits to produce oligomers showing kinetic parameters comparable to MAT II purified from several tissues. According to isothermal titration calorimetry data and densitometric scanning of the stained hetero-oligomer bands on denatured gels, the composition of these oligomers is that of a hetero-trimer with α2 dimers associated to single β subunits. Additionally, the regulatory subunit is able to bind NADP^+^ with a 1∶1 stoichiometry, the cofactor enhancing β to α2-dimer binding affinity. Mutants lacking residues involved in NADP^+^ binding and N-terminal truncations of the β subunit were able to oligomerize with α2-dimers, although the kinetic properties appeared altered. These data together suggest a role for both parts of the sequence in the regulatory role exerted by the β subunit on catalysis. Moreover, preparation of a structural model for the hetero-oligomer, using the available crystal data, allowed prediction of the regions involved in β to α2-dimer interaction. Finally, the implications that the presence of different N-terminals in the β subunit could have on MAT II behavior are discussed in light of the recent identification of several splicing forms of this subunit in hepatoma cells.

## Introduction

Mammalian methionine adenosyltransferase (MAT) II is the only hetero-oligomer identified to date in the MAT family of enzymes (MAT, EC 2.5.1.6) that catalyze S-adenosylmethionine (AdoMet) synthesis using methionine and ATP as substrates [1]. This isoenzyme is composed of catalytic (α2) and regulatory (β) subunits encoded by the *MAT2A* and *MAT2B* genes, respectively. The precise hetero-oligomer association state remains under debate, although it has been postulated to be tetrameric (α2β)_2_, according to the results obtained from gel filtration chromatography and sedimentation velocity experiments [2]–[4]. Mammalian MAT isoenzymes, including MAT II, show dependency on Mg^2+^, stimulation by K^+^ and tripolyphosphatase activity. However, they differ in their affinities for methionine, MAT II exhibiting the highest, followed by MAT I and MAT III. The V_max_ are also dissimilar, following the opposite trend [1]. Their response to AdoMet varies; MAT I (α1_4_) and MAT II are inhibited by the reaction product, whereas AdoMet activates MAT III (α1_2_) [1].

Classical studies identified MAT II in the cellular cytosol of extrahepatic and tumor cells, whereas MAT I and III were described as the hepatic isoenzymes [1], [5]. Development of liver cirrhosis and hepatocellular carcinoma (HCC) induces a switch in the expression of the isoenzymes in which *MAT1A* (encoding α1) expression is reduced and that of *MAT2A* increased [1], [6]. Given the differences in affinity and V_max_ of these isoenzymes, this expression change leads to a reduction in the levels of AdoMet, the main methyl donor for cellular transmethylations, and among them some epigenetic modifications. Further confirmation of the importance that maintenance of AdoMet levels has for the cell was obtained upon production of knockout mice for *MAT1A* and *GNMT* (glycine N-methyltransferase). These mice exhibit low and high AdoMet concentrations, respectively, and spontaneously develop HCC [7], [8]. Recent reports have shown both α1 and α2 proteins in the nuclear compartment, where AdoMet production was measured [9], [10]. Nuclear accumulation of MAT α1 correlated with histone 3 K27 trimethylation, whereas α2 was identified as a corepressor of MafK transcription factor. All these data together suggest that AdoMet synthesis is carried out close to where it is needed, hence the enzymes move to the nuclear compartment to provide methyl groups for epigenetic modifications [9]–[11].

**Figure 1:**
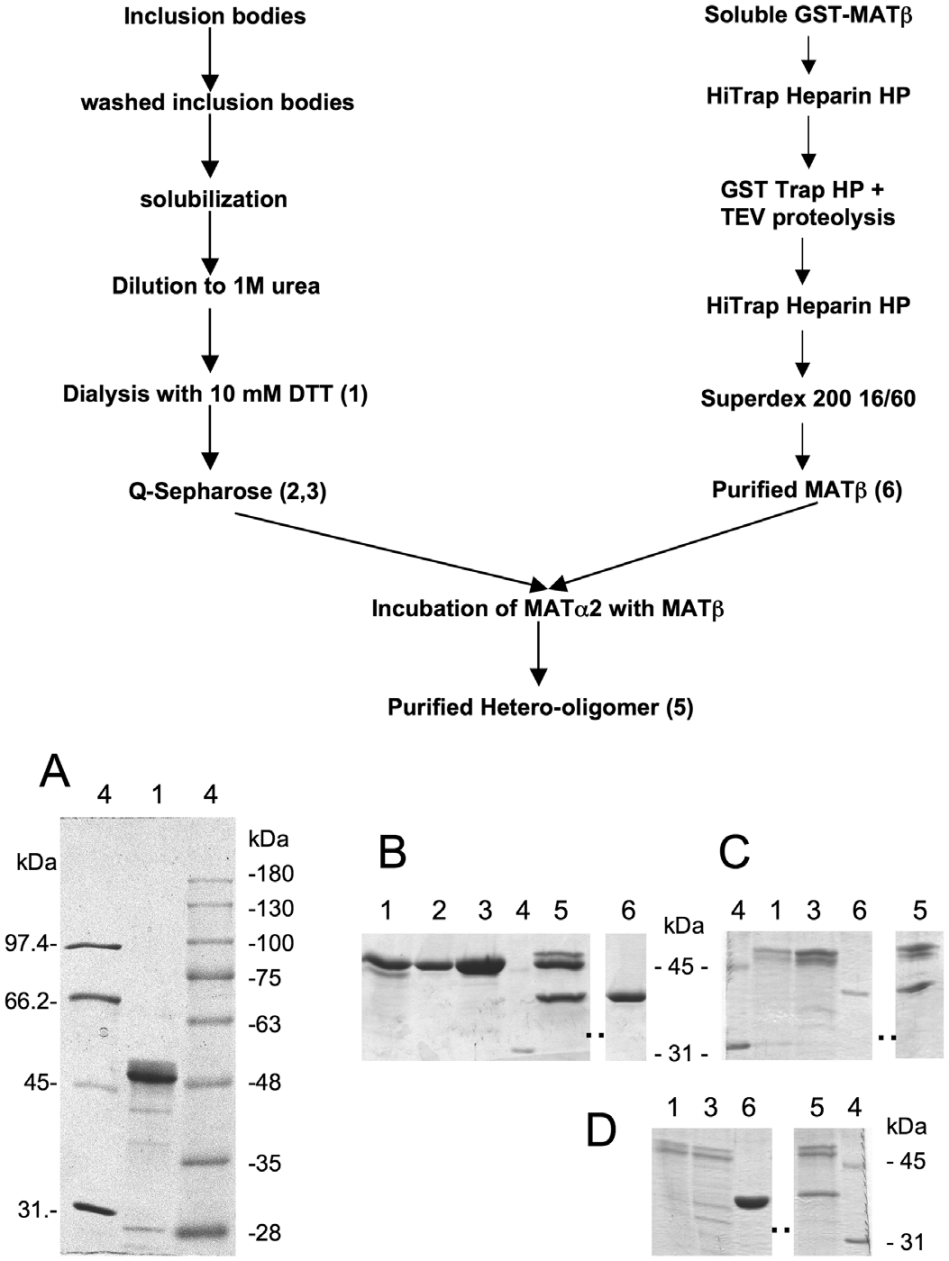
Production of recombinant proteins and purification steps. The upper part of the figure shows a scheme of the purification steps followed to prepare the three types of hetero-oligomers obtained in this work. The numbers indicate the corresponding lane of the representative stained gels shown below. Panel A shows a representative SDS-PAGE gel used for molecular mass estimation, including two sets of standards as indicated in the Materials and Methods section. Samples (30 µl) of key purification steps to produce wild type (B), mutated (C) and truncated MAT II (D) were prepared under standard reducing conditions for SDS-PAGE electrophoresis. Gel lanes correspond to: (1) refolded α2 (10 µg); (2) Q-Sepharose collected peak (10 µg); (3) concentrated Q-Sepharose peak (25 µg); (4) standards; (5) purified hetero-oligomer (10 µg); and (6) purified wild type (10 µg), mutant (5 µg) or truncated β subunit (10 µg). Panels B–D show only the relevant sections of the stained gels for each type of purification; the positions for the 45 and 31 kDa protein standards are indicated on the side of the gels. Dots indicate places where gel lanes have been cropped for clarity.

The role of the regulatory subunit has been explored mostly in lymphocytes and also after overexpression in other cell lines and bacteria [1]. The results obtained indicated that binding of the β subunit to α2 enhances the affinity for methionine (3.3 µM vs. 80 µM) and decreases sensitivity to AdoMet inhibition [2], [12]. *MAT2B* expression has been shown to increase during liver cirrhosis and HCC development [1], [13], but its expression not always follows that of *MAT2A.* Early stages of Wilson disease in the Long Evans Cinnamon rat model showed the *MAT1A* to *MAT2A* hepatic switch, but a strong reduction in *MAT2B* expression [14]. This effect was also observed in hepatoma H35 cells treated with copper, and was prevented by the addition of buthionine sulfoximine, an inhibitor of glutathione synthesis.

**Figure 2:**
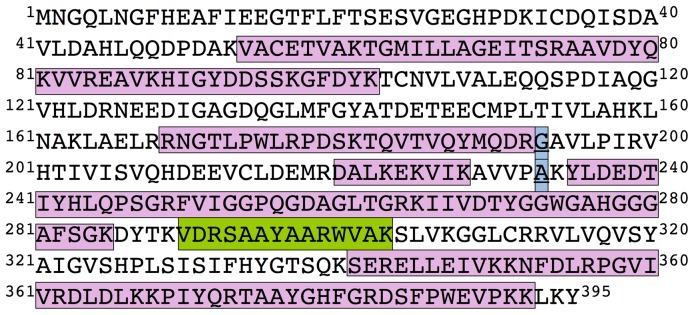
MAT α2 sequence identified by peptide mass fingerprint using LC-MS/MS. The figure shows the amino acid sequence of human MAT α2, where the peptides identified by mass spectrometry are indicated. Blue squares highlight the two mutations detected in the ORF (underlined). Pink squares indicate the peptides identified with high scores in both α2 and α2′ bands. The green square indicates a peptide identified in both bands, but showing higher score for α2′ than for the α2 band. The sequence coverage is ∼50% of the total protein sequence.

**Table 1:** Results of peptide mass fingerprint of MAT α2 bands.

RESULTS FOR α2					RESULTS FOR α2′				
Mass Mr	Dev	Range	P	Sequence	Mass Mr	Dev	Range	P	sequence
					561.231	−0.039	308–312	0	GGLCR
600.314	−0.046	164–168	0	LAELR	600.307	−0.052	164–168	0	LAELR
628.240	−0.045	98–102	0	GFDYK	628.292	0.006	98–102	0	GFDYK
708.299	−0.056	293–299	0	SAAYAAR	708.332	−0.023	293–299	0	SAAYAAR
					717.280	0.009	308–313	0	GGLCRR
724.432	−0.028	193–199	0	GAVLPIR	724.429	−0.031	193–199	0	GAVLPIR
756.460	−0.001	164–169	1	LAELRR	756.419	−0.042	164–169	1	LAELRR
					793.368	−0.029	75–81	0	AAVDYQK
803.407	−0.058	368–373	0	KPIYQR					
895.390	−0.050	286–292	1	DYTKVDR	895.410	−0.030	286–292	1	DYTKVDR
978.418	−0.049	374–382	0	TAAYGHFGR	978.428	−0.039	374–382	0	TAAYGHFGR
1020.350	−0.102	89–97	0	HIGYDDSSK	1020.410	−0.041	89–97	0	HIGYDDSSK
1103.435	−0.094	383–391	0	DSFPWEVPK	1103.466	−0.062	383–391	0	DSFPWEVPK
1231.541	−0.083	383–392	1	DSFPWEVPKK	1231.535	−0.089	383–392	1	DSFPWEVPKK
1284.671	−0.059	352–362	0	NFDLRPGVIVR					
1360.657	−0.082	62–74	0	TGMILLAGEITSR	1360.620	−0.118	62–74	0	TGMILLAGEITSR
1367.596	−0.054	182–192	0	TQVTVQYMQDR	1367.523	−0.128	182–192	0	TQVTVQYMQDR
1382.681	−0.050	170–181	0	NGTLPWLRPDSK	1382.636	−0.094	170–181	0	NGTLPWLRPDSK
1383.674	0.029	182–192	0	TQVTVQYMQDR	1383.611	−0.034	182–192	0	TQVTVQYMQDR
1387.732	−0.050	363–373	1	DLDLKKPIYQR	1387.663	−0.119	363–373	1	DLDLKKPIYQR
1412.771	−0.054	351–362	1	KNFDLRPGVIVR	1412.722	−0.103	351–362	1	KNFDLRPGVIVR
1443.718	−0.029	250–264	0	FVIGGPQGDAGLTGR	1443.642	−0.105	250–264	0	FVIGGPQGDAGLTGR
1571.809	−0.033	250–265	1	FVIGGPQGDAGLTGRK	1571.741	−0.101	250–265	1	FVIGGPQGDAGLTGRK
1630.673	−0.054	89–102	1	HIGYDDSSKGFDYK	1630.622	−0.105	89–102	1	HIGVDDSSKGFDYK
1805.855	−0.003	235–249	0	YLDEDTIYHLQPSGR	1805.795	−0.063	235–249	0	YLDEDTIYHLQPSGR
1948.944	0.000	266–285	0	IIVDTYGGWGAHGGGAFSGK	1948.806	−0.137	266–285	0	IIVDTYGGWGAHGGGAFSGK
					2064.029	0.043	374–391	1	TAAYGHFGRDSFPWEVPK
2077.058	0.020	265–285	1	KIIVDTYGGWGAHGGGAFSGK	2076.911	−0.127	265–285	1	KIIVDTYGGWGAHGGGAFSGK
					2191.913	−0.167	374–392	2	TAAYGHFGRDSFPWEVPKK
					2408.073	−0.073	200–219	0	VHTIVISVQHDEEVCLDEMR
2456.189	0.013	266–289	1	IIVDTYGGWGAHGGGAFSGKDYTK	2456.043	−0.133	266–289	1	IIVDTYGGWGAHGGGAFSGKDYTK
					2562.305	0.019	103–125	0	TCNVLVALEQQSPDIAQGVHLDR
2584.297	0.026	265–289	2	KIIVDTYGGWGAHGGGAFSGKDYTK	2584.246	−0.025	265–289	2	KIIVDTYGGWGAHGGGAFSGKDYTK

Purified refolded-α2 was loaded in SDS-PAGE gels, the two bands (α2 and α2′) observed after staining were excised and digested with trypsin. Peptides were desalted and subjected to MALDI-TOF to obtain the corresponding mass fingerprint. Database searches were performed with MASCOT and successful protein identification was considered when p<0.05.

Catalytic α2 subunits preserve the average length (∼390 residues), the conserved sequence blocks (including substrate binding motifs) and the folding (pdb code 2P02) that characterize this protein family [15], [16]. The crystal structure of α2 monomers shows the typical three-domain organization formed by nonconsecutive stretches of the sequence exhibited by α subunits from *Bacteria* to *Eukarya*. Association of α2 subunits into dimers occurs through a flat hydrophobic surface, and amino acids from both monomers constitute the two catalytic sites that locate between subunits opposed one to another. In contrast, the β subunit is a non-related protein, for which four splicing forms (named V1, V2, V2a, V2b) have been recently identified in hepatoma cells [17]. The highest expression levels correspond to V1 and V2 forms that differ in the exon 1 used during transcription, thus leading to proteins that differ in their N-terminal sequences. Most studies carried out to date refer to the V1 splicing form of 334 residues, whose crystal structure has been recently obtained alone and in complex with NADP^+^ (pdb codes 2YDY and 2YDX, respectively). The V1 sequence exhibits 28% homology to bacterial enzymes that catalyze reduction of TDP-linked sugars, several nucleoside-diphosphate sugar epimerases and other proteins involved in polysaccharide synthesis [1], [15], and hence the protein was classified as an oxidoreductase belonging to the PFAM 04321 family.

**Figure 3:**
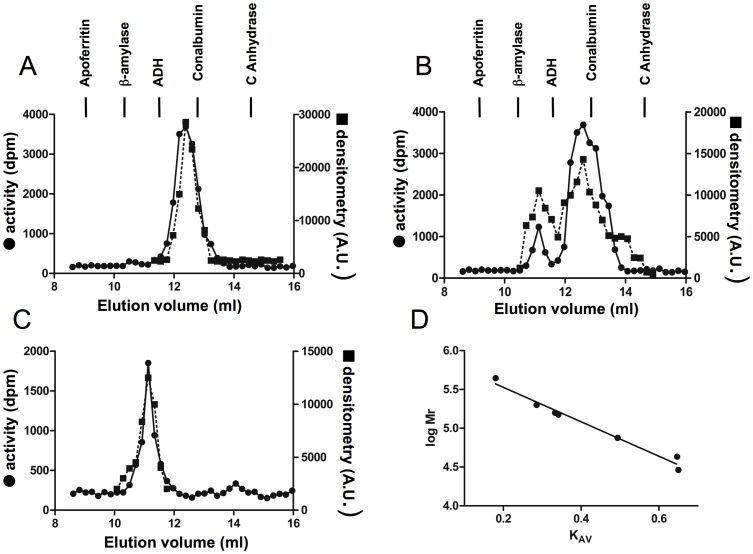
Analytical gel filtration chromatography of MAT proteins. The figure shows gel filtration chromatography profiles of purified MAT forms followed by activity measurements (•) and densitometric scanning of Dot Blots incubated with anti-MATα2 (▪). The elution behavior of the α2 subunit is depicted in panels A (50 µg) and B (200 µg), and that of the MAT II hetero-oligomer including the wild type β subunit appears in panel C. Panel D shows a representative regression line for the standards used in column calibration [log M_r_ = (−2.085×K_AV_)+5.903]. The elution volume of representative standards is indicated by a vertical bar and specified in the Materials and Methods section.

Most of the pathological processes in which MAT II is involved share production of a certain degree of oxidative stress that may be implicated in MAT II regulation as described for MAT I/III [1], [18]. The occurrence of NADP^+^ binding to the β subunit, postulated by the analysis of its sequence, and observed in one of the solved crystal structures (2YDX), also suggests this possibility. However, in this case the subunit involved might be β subunit rather than α2. Hence, the aims of this study were two, first to establish the MAT II association state and second to analyze the role of NADP^+^ binding on MAT II.

**Table 2:** MAT activity data of α2 homo- and hetero-oligomers.

	V_max_ (nmol/min/mg)			
	− NADP^+^	p value vs. MAT II	+ NADP^+^	p value +/− NADP^+^
**α2**	187.38±27.37	0.01*	–	–
**MAT II**	33.95±7.52	–	36.53±10.06	0.74
**Mutant MAT II**	45.76±11.02	0.16	58.73±20.30	0.26
**Truncated MAT II**	58.00±2.36	0.01*	43.56±7.51	0.04*

The table shows V_max_ data of α2 homo-dimers and hetero-oligomers composed by: α2 and wild type β (MAT II); α2 and Y159F/K163A-β (mutant MAT II); and α2 and ΔS16 β subunit (truncated MAT II). The results shown are the mean ± SD of a minimum of three independent experiments carried out in triplicate that were considered significant when p≤0.05 (*).

**Table 3:** Methionine kinetics of α2 homo- and hetero-oligomers.

	S_0.5_^Met^ (µM)			
	− NADP^+^	p value vs. MAT II	+ NADP^+^	p value +/− NADP^+^
**α2**	395.44±60.12	0.01*	–	–
**MAT II**	35.19±29.43	–	29.74±2.16	0.82
**Mutant MAT II**	84.00±58.24	0.27	70.51±5.93	0.79
**Truncated MAT II**	152.33±1.04	0.02*	132.42±23.81	0.21

The table shows the calulated S_0.5_ values for methionine of α2 homo-dimers and hetero-oligomers composed by: α2 and wild type β (MAT II); α2 and Y159F/K163A-β (mutant MAT II); and α2 and a ΔS16 β subunit (truncated MAT II). S_0.5_ refers to the concentration of substrate at which half the V_max_ is achieved. The results shown are the mean ± SD of a minimum of three independent experiments carried out in triplicate and were considered significant only when p≤0.05 (*).

**Table 4:** ATP kinetics for α2 homo- and hetero-oligomers.

	S_0.5_^ATP^ (µM)			
	− NADP^+^	p value vs. MAT II	+ NADP^+^	p value +/− NADP^+^
**α2**	254.40±66.18	0.01*	–	–
**MAT II**	67.49±15.87	–	74.52±12.56	0.53
**Mutant MAT II**	124.53±32.20	0.01*	142.37±32.21	0.48
**Truncated MAT II**	150.85±81.37	0.05*	154.34±34.86	0.95

The table shows the calulated S_0.5_ values for ATP of α2 homo-dimers and hetero-oligomers composed by: α2 and wild type β (MAT II); α2 and Y159F/K163A-β (mutant MAT II); and α2 and a ΔS16 β subunit (truncated MAT II). S_0.5_ refers to the concentration of substrate at which half the V_max_ is achieved. The results shown are the mean ± SD of a minimum of three independent experiments carried out in triplicate and were considered significant only when p≤0.05 (*).

## Materials and Methods

### 1. Constructions and Site Directed Mutagenesis

The cDNAs of human *MAT2A* and *MAT2B* were obtained by RT-PCR using the Superscript One-Step RT-PCR kit (Invitrogen) and total lymphocyte RNA kindly provided by Dr. López Trascasa of the Hospital Universitario La Paz (Madrid, Spain). In both cases cDNA synthesis was performed at 50°C for 30 min. PCR was carried out for 35 cycles with 1 min annealing steps at 60°C and 50°C for MAT2A and MAT2B, respectively. The primers for cloning into pT7.7 included NdeI and EcoRI sites that appear underlined; their sequences were: 5′-GGGAATTCCATATGAACGGACAGCTCAACGGCTT-3′ (sense) and 5′-CGGA ATTCAGCCTACGCCAACAAGTCTGGGGA-3′ (reverse) for MAT2A and 5′-GGGA ATTCCATATGGTGGGGCGGGAGAAAGAGCTCTCT-3′ (sense) and 5′-CGGAAT TCACAGGGCATGACTGCCCTTTAGT-3′ (reverse) for MAT2B. Sequences were verified by automatic sequencing at the Genomic Service of the Instituto de Investigaciones Biomédicas “Alberto Sols”. All the MAT2A ORFs cloned included two substitutions rendering α2 subunits with A233V and G193S mutations. The resulting plasmids were named pT7.7-MAT2A and pT7.7-MAT2B.

**Figure 4:**
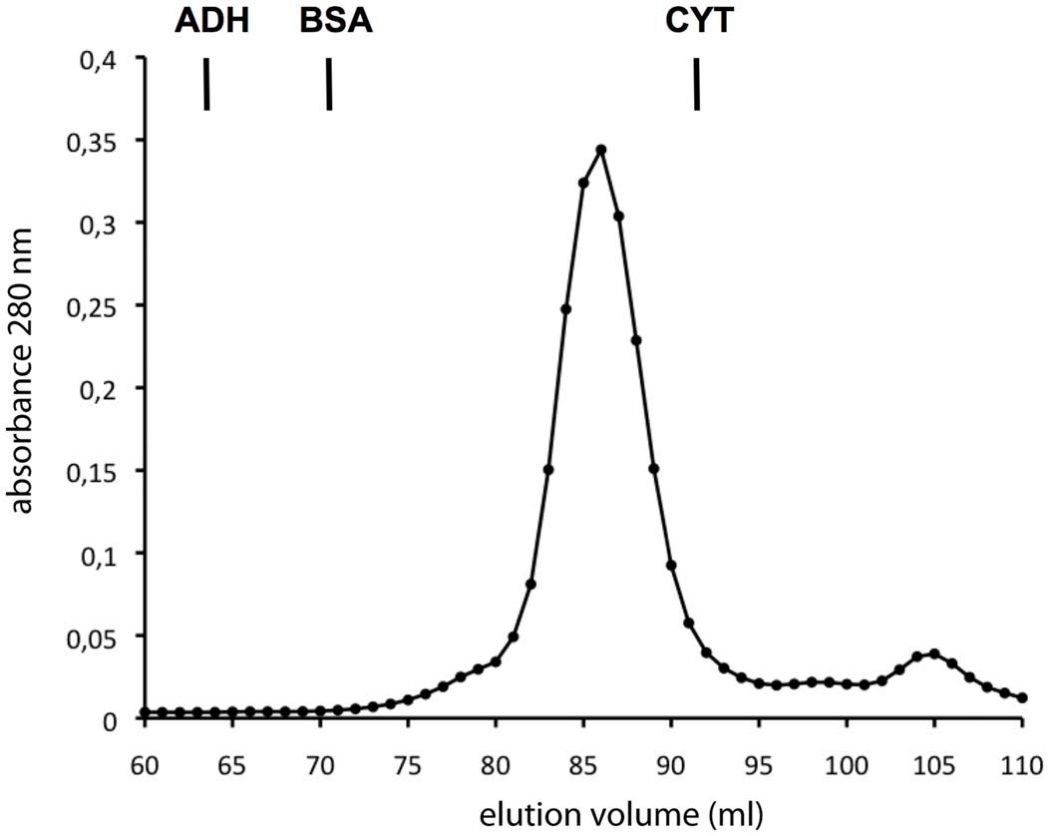
Gel filtration chromatography of the β subunit. The purified wild type β subunit was analyzed on a Superdex 200 16/60 gel filtration column and the elution followed by A_280_. Vertical bars indicate the elution position of relevant standards. The elution volume of all the markers used was: dextran blue (2000 kDa) 39.4 ml; apoferritin (443 kDa) 52.3 ml; β-amylase (200 kDa) 59.5 ml; alcohol dehydrogenase (150 kDa) 64.3 ml; bovine serum albumin (66.2 kDa) 71.6 ml; and cytochrome *c* (12.4 kDa) 93 ml.

**Figure 5:**
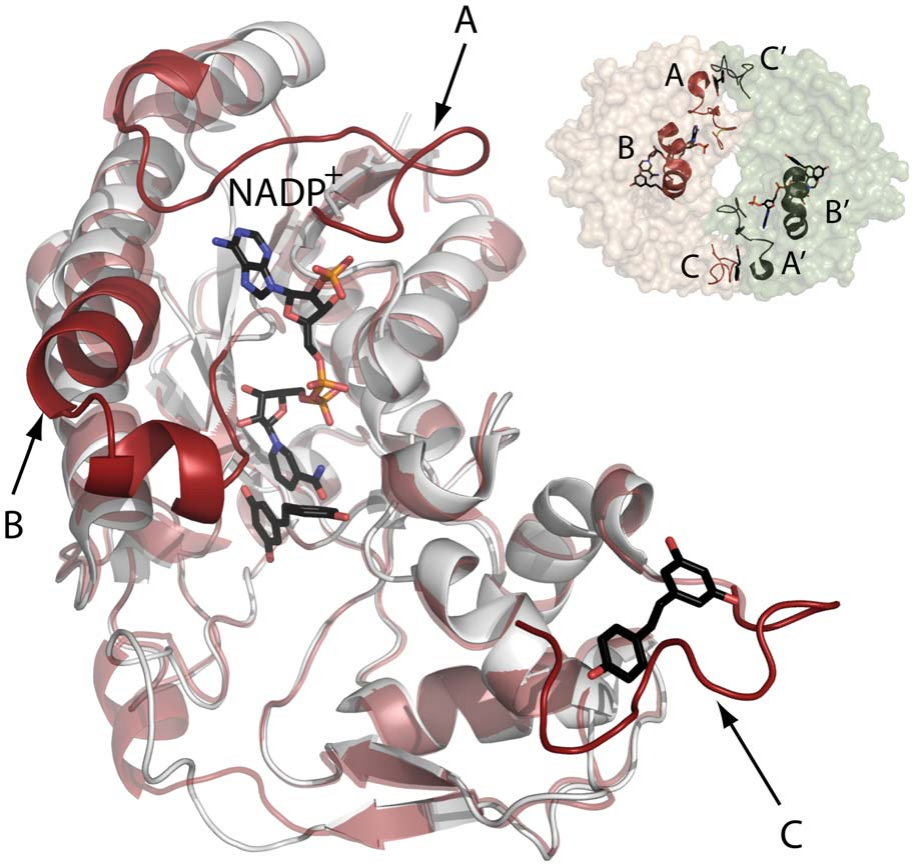
Comparison of the structures of human β subunits in free and NADP^+^ bound states. The figure depicts the structure of free β subunits (white; 2YDY) and that of the NADP^+^ bound state (red; 2YDX). Cofactor and resveratrol molecules are shown as sticks with coloured carbon (black), nitrogen (red), phosphorus (orange) and oxygen (blue) atoms. Three regions not visible in the apoenzyme and ordered upon NADP^+^ binding are highlighted: F60-A77 (A), A95-N113 (B), and D325-F333 (C); only A and B regions are linked to NADP^+^. On the right the surface of the β dimer is shown as found in the NADP^+^ bound crystal structure, illustrating location of A and C regions in the dimer interface.

MAT2A was also cloned into the pOPTH vector, to produce the corresponding Met-Ala-(His)_6_-tagged protein. For this purpose, the ORF sequence was amplified using pT7.7-MAT2A as template and KOD polymerase (Novagen). Elimination of a BamHI internal site required a first PCR step that generated two fragments. Primers used for fragment 1 production were 5′-GGAGATATACATATGAACGGACAGCTCAACGG C-3′ (sense) and 5`-CTTTGGCATCAGGGTCCTGCTGAAGG-3′ (reverse), whereas fragment 2 synthesis utilized primers 5′-CCTTCAGCAGGACCCTGATGCCAAAG-3′ (sense) and 5′-CGCGGATCCTCAATATTTAAGCTTTTTGG-3′ (reverse). A second PCR step utilized a mixture of both fragments as templates together with the primers 5′-GGAGATATACATATGAACGGACAGCTCAACGGC-3′ (sense) and 5′-CGCGGAT CCTCAATATTTAAGCTTTTTGG-3′ (reverse) including NdeI and BamHI restriction sites (underlined), respectively. The final PCR program included 20 cycles with 1 min annealing steps at 55°C. The resulting plasmid was named pOPTH-MAT2A.

**Figure 6:**
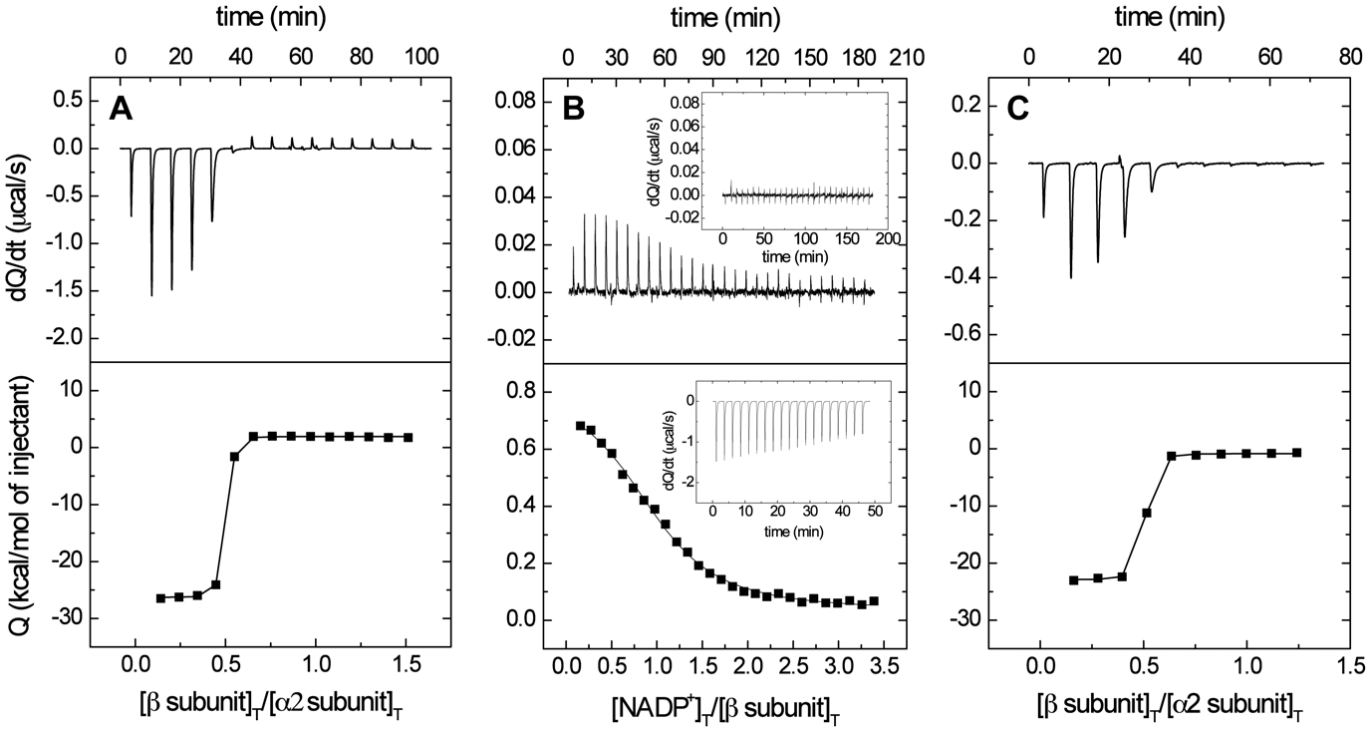
Isothermal titration calorimetry of MAT subunits in the presence or absence of NADP^+^. The figure shows representative titration experiments carried out with the wild type regulatory β subunit to characterize binary and tertiary binding with α2 subunits and/or NADP^+^. Panel A depicts titration of α2 (13.4 µM in the cell) and β subunits (190 µM in the syringe). Panel B shows titration of the β subunit (9.8 µM in the cell) and NADP^+^ (155 µM in the syringe); the insets show titrations using the β subunit (20 µM) and 300 µM NAD^+^ (top) and the Y159F/K163A-β subunit (20 µM) and 330 µM NADP^+^ (bottom). The very low affinity observed for the mutant precluded a precise estimation of the binding affinity, and hence only a lower limit for the dissociation constant could be determined. Panel C illustrates titration of α2 (4.3 µM in the cell) and β subunits (62.4 µM in the syringe) in the presence of NADP^+^ (300 µM in both the cell and the syringe). All measurements were performed at 25°C as described in the Materials and Methods section.

**Table 5:** Thermodynamic parameters for MAT II interactions.

	NADP^+^→β	α2→ β	α2→ β^a^
**K_a_ (M** ^−**1**^ **)**	5.8±0.6×10^5^	2.7±0.3×10^8^	>4.0±0.4×10^8^
**K_d_ (M)**	1.7±0.2×10^−6^	3.7±0.4×10^−9^	<2.5±0.3×10^−9^
**ΔG (kcal/mol)**	−7.9±0.1	−11.5±0.1	<−11.7±0.1
**ΔH (kcal/mol)**	0.8±0.2	−28.3±0.2	−22.2±0.2
**−TΔS (kcal/mol)**	−8.7±0.2	16.8±0.2	<10.5±0.2
**n**	0.99±0.02	0.45±0.02	0.45±0.02

The purified MAT β subunit was titrated with NADP^+^ or the α2 subunit (in the presence or absence of NADP^+^) using ITC and the parameters included in the table were obtained. Relative errors in *K*_a_ and *K*_d_ were typically 20%; absolute errors in ΔH and -TΔS were 0.3 kcal/mol.

a Data in the presence of NADP^+^ (300 µM).

The MAT2B ORF was also cloned into the pOPTG vector to produce a GST-MAT β fusion protein (linker sequence ENLYFQGSH). For this purpose, the sequence was amplified from pT7.7-MAT2B using KOD polymerase and the primers 5′-GGAG ATATACATATGGTGGGGCGGGAGAAAGAGC-3′ (sense) and 5′-CGTGACGGAA GCTTCTAATGAAAGACCGTTTGTCTCC-3′ (antisense) including NdeI and HindIII restriction sites (underlined), respectively. The PCR program included 20 cycles with 1 min annealing steps at 55°C. The resulting plasmid was named pOPTG-MAT2B. Truncated MAT2B constructs were obtained by amplification of the desired sequence from the pT7.7-MAT2B vector using the same PCR conditions, the above described reverse primer and sense primers containing a NdeI restriction site for cloning into pOPTG. Primer sequences (sense only) for ΔS16 and ΔR29 were 5′-GGAGATATAC ATATGAGCTGTCGGCTGGTGGAGG-3′ and 5′-GGAGATATACATATGAGGAG GGTTCTGGTTACTGG-3′, respectively.

**Figure 7:**
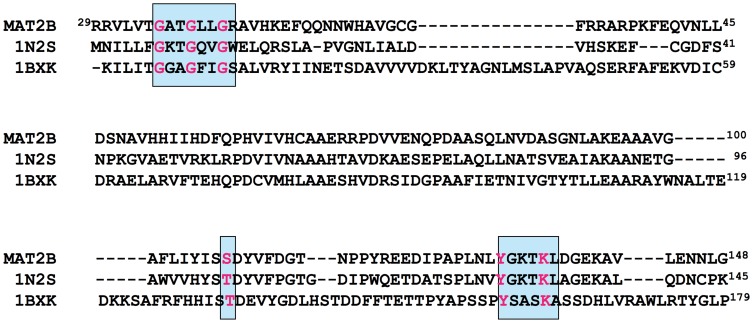
Sequence comparison of the β subunit (V1 form) with relevant members of the RED family. The figure shows an alignment of sequences for Dtdp-6-deoxy-L-lyxo-4-hexulose reductase (1N2S), Dtdp-glucose 4,6-dehydratase (1BXK) and the β regulatory subunit of MAT II. Conserved residues of the GXXGXXG phosphate binding motif and the catalytic triad (SX_N_YXXXK) appear in blue.

**Figure 8:**
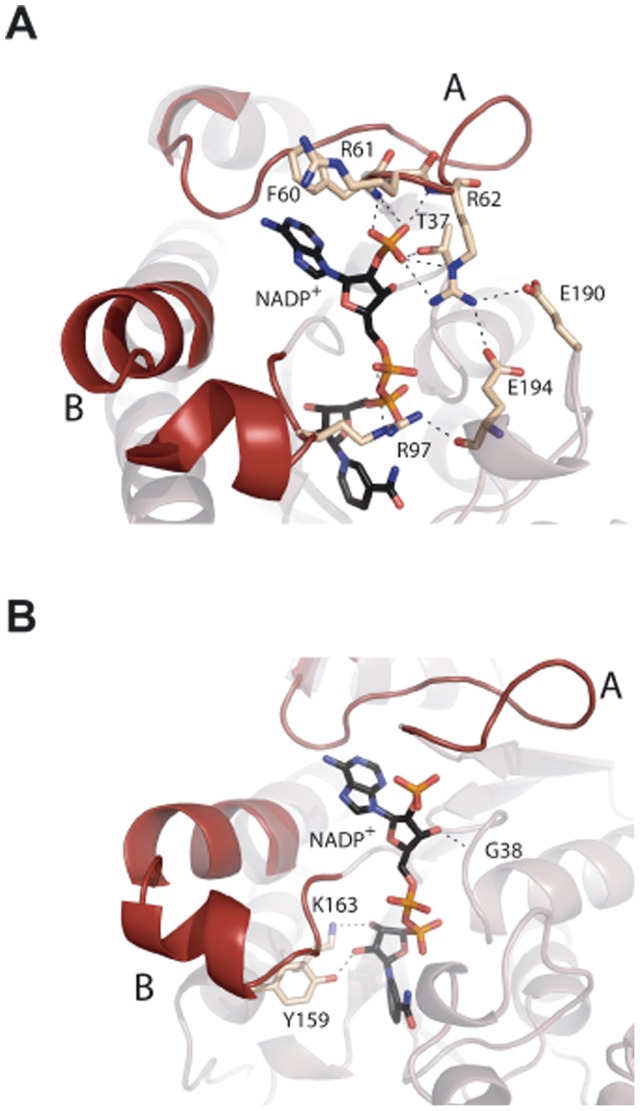
The NADP^+^ binding site in the β subunit. The figure shows the structure of the NADP^+^ site with the protein residues shown as wheat sticks. Colour codes are as in Figure 4. Panel A depicts recognition of the phosphate moieties, showing that segment A is involved in NADP^+^/NAD^+^ discrimination by linking the additional phosphate group. This phosphate is linked to R62 (region A) which establishes a strong network of atomic interactions that connects to R97 (region B), conforming the NADP^+^ binding site. Panel B shows a detail of the residues that bind NADP^+^ and have been mutated in this work.

Site-directed mutagenesis was carried out in pOPTG-MAT2B using the QuikChange method (Stratagene) and the primers (changes underlined): 5′-GGTTAC TGGTGCCACTGTGCTTCTTGGCAGAGC-3′ and its complementary (G38V); 5′-CC TTAAATTTGTTTGGCAAAACAGCATTAGATGGAG-3′ and its complementary (Y159F/K163A). Sequences of all the plasmids were verified by automatic sequencing.

**Figure 9:**
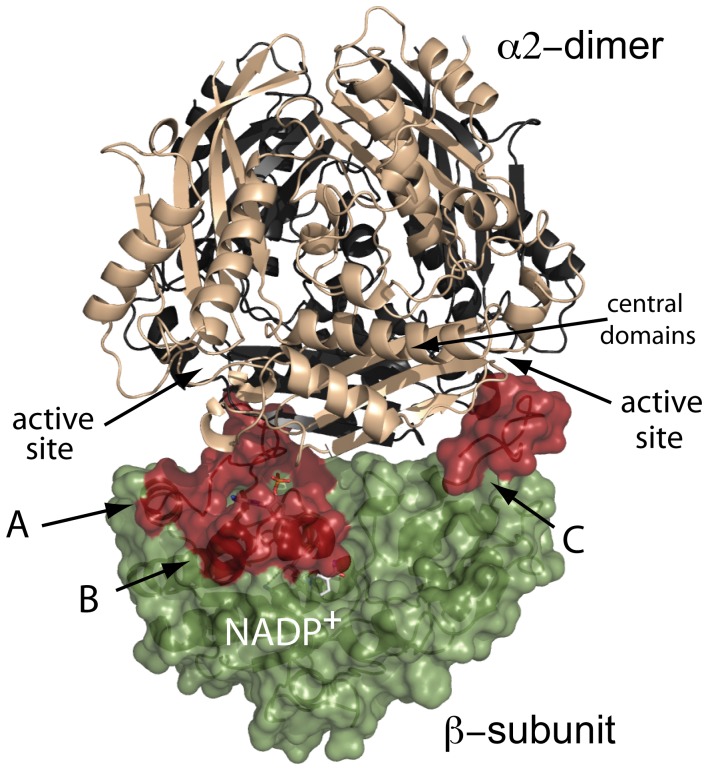
Structural model of the MAT II trimer. A putative model of the MAT II hetero-oligomer was obtained with ClusPro using available coordinates for α2 (2P02) and β (2YDX) subunits. The α2 subunits in the dimer are shown as cream and black cartoons. Each α2 monomer consists of N-terminal, central and C-terminal domains, both subunits being related by a 2-fold symmetry axis. The β subunit is represented as a green surface, with A, B and C regions highlighted in red. The hetero-oligomer interface is formed by central domains of both α2 monomers and regions A, B and C from the regulatory β subunit. Only models displaying β interacting to α2 central domains, therefore located on its 2-fold axis, would be consistent with a 2∶1 stoichiometry in the hetero-oligomer.

### 2. MAT α2 Overexpression and Purification

Competent BL21 (DE3) Codon Plus were transformed with pT7.7-MAT2A and grown in LBA medium at 37°C until A_600_ = 0.3–0.4 when 0.5 mM IPTG (Ambion) was added and the culture transferred to 27°C for 20 hours. The refolded-α2 protein was obtained from inclusion bodies using a procedure adapted from López-Vara et al. [19]. The changes refer to solubilization of the washed inclusion bodies with urea 8 M for 4 hours at 10°C and dilution to 1 M urea at the first refolding step. Purification was carried out as previously described for MAT I/III [19].

**Figure 10:**
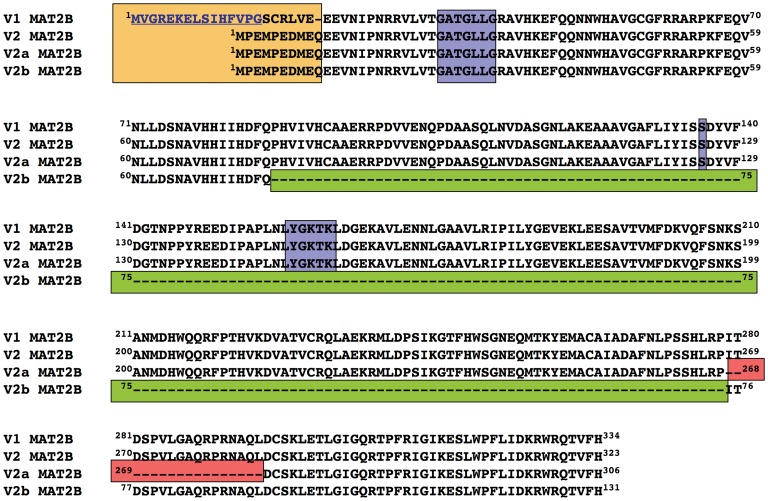
Sequence alignment of regulatory subunit splicing forms. Sequences of the four splicing forms reported to date for the regulatory β subunit of MAT II in hepatoma cells were aligned against the most abundant V1 form (334 residues). The figure shows underlined the residues truncated in the ΔS16 protein used in this study. In addition, several differences among splicing forms are also indicated: the N-terminal differences (orange), the sequence lacking in V2a (pink) and V2b (green), and the putative NADP^+^ binding motifs (blue).

Alternatively, competent BL21 (DE3) cells were transformed with pOPTH-MAT2A and grown in 2TY medium at 37°C until A_600_ = 0.6. In this case IPTG induction was carried out 18 hours at 16°C. The cell pellet was resuspended in 20 mM sodium phosphate pH 7.5 10 mM MgSO_4_, 5 mM DTT, 100 mM KCl (buffer A) and lysed in a French press. The soluble fraction was loaded into a His Trap HP 5 ml column (GE Healthcare) equilibrated in buffer A, followed by a washing step with buffer A containing 15 mM imidazol (50 ml). Elution was performed with a gradient (120 ml) from 15 to 300 mM imidazol in buffer A and 4 ml fractions collected. Samples of these fractions were loaded on SDS-PAGE gels that were stained to detect Met-Ala-(His)_6_-α2. Fractions containing the fusion protein were pooled and loaded on a 5 ml Q HP column (GE Healthcare) equilibrated in 20 mM Tris/HCl pH 8, 10 mM MgSO_4_, 5 mM DTT (buffer B). Following washing with buffer B (50 ml), elution was performed with a gradient (120 ml) from 0 to 1 M KCl in buffer B and 4 ml fractions collected. Fractions containing tagged-α2 were pooled, concentrated and loaded on a Superdex 200 16/60 column equilibrated and run in buffer B containing 100 mM KCl at 1 ml/min. Fractions (1 ml) were collected and those containing the purified protein were pooled.

### 3. MAT β Overexpression and Purification

Competent BL21 Rosetta (DE3) pLysS were transformed with pOPTG-MAT2B and grown in 2TY medium at 37°C until A_600_ = 0.7. IPTG (0.5 mM) was added and the culture was grown for 16 hours at 16°C. The cell pellet was resuspended in 50 mM Tris/HCl pH 7.5 containing 2 mM DTT (buffer C), lysed in a French press and the soluble fraction loaded on a HiTrap Heparin HP 5 ml column (GE Healthcare) equilibrated in buffer C. The column was washed first with 50 ml buffer A plus 10% (v/v) Triton X-100 (Merck), followed by 50 ml of buffer C. Elution was performed with a 120 ml gradient from 0 to 1 M NaCl in buffer C and 4 ml fractions collected. GST-MATβ was detected by SDS-PAGE staining of fraction samples. The fractions containing the fusion protein were pooled and loaded onto a GSTrap HP 5 ml column (GE Healthcare) equilibrated in buffer C containing 200 mM NaCl at 4°C (buffer D). After washing with buffer D (100 ml), cleavage was performed with home made TEV protease (protein:TEV mass ratio 80∶1) that was loaded onto the column and left at 4°C o/n. The eluted β subunit (retaining the GSH linker sequence) was collected, diluted 3-fold with buffer D and reloaded on the heparin column to remove TEV protease. The purified β protein was pooled and made 50% (v/v) with glycerol for storage at −20°C. Gel filtration chromatography of the β protein was carried out on a Superdex 200 16/60 column equilibrated and run in buffer 20 mM Tris/HCl pH 8, 100 mM KCl, 10 mM Mg_2_SO_4_ at 1 ml/min and 1 ml fractions were collected. All mutants and truncated forms were obtained following the same protocol as the wild type protein.

### 4. In vitro Production and Isolation of MAT II Hetero-oligomers

Purified refolded-α2 and β subunits (wild type, mutant or truncated) were dialyzed separately against 50 mM Tris/HCl pH 8, 10 mM MgSO_4_, 50 mM KCl (buffer E). Dialyzed samples were concentrated by ultrafiltration using AMICON PM-10 membranes to obtain protein concentrations of ∼1 mg/ml. Equimolar amounts of α2 and each β protein were mixed together and incubated for 1 hour at 4°C before gel filtration chromatography on a Biogel A 1.5 m column (1.5×90 cm; BioRad) equilibrated in buffer E. Elution was carried out at 10 ml/h using buffer E and 3 ml fractions were collected. The peak containing MAT II hetero-oligomers was identified by measuring A_280_ and MAT activity and collected. The presence of both subunits was confirmed by SDS-PAGE of the fractions.

### 5. Determination of MAT Activity and Kinetics

Activity assays were carried out at 37°C as previously described [20], using 0.05 mg/ml protein concentrations and the standard MAT reaction mixture containing 5 mM methionine (Sigma) and 5 mM ATP (Sigma) in a final volume of 250 µl. Kinetics were performed using reaction mixtures containing 1–600 µM of the amino acid and 5 mM ATP (methionine kinetics) or 1–1500 µM of the nucleotide and 5 mM methionine (ATP kinetics), NADP^+^ (Sigma) 50 µM was added to the protein mixture when required.

### 6. Analytical Gel Filtration Chromatography

Samples of refolded-α2 subunits and MAT II complexes (100 µl containing a minimum of 50 µg) were injected onto a Superose 12 10/300 GL gel filtration column (GE Healthcare) equilibrated and run in 20 mM Tris/HCl pH 8, 10 mM MgSO_4_, 150 mM KCl. The flow rate was 0.3 ml/min and 210 µl fractions were collected. MAT detection by activity measurements (100 µl) and Dot Blot using 1∶1000 (v/v) chicken anti-MATα2 (Abcam) were performed as previously described [21]. The protein standards (GE Healthcare and Sigma) used and their elution volumes were as follows: Dextran Blue (2000 kDa), 7.66 ml; apoferritin (443 kDa), 9.14 ml; β-amylase (200 kDa), 10.7 ml; alcohol dehydrogenase (150 kDa), 11.27 ml; aldolase (158 kDa), 11.1 ml; conalbumin (75 kDa), 12.89 ml; ovalbumin (43 kDa), 14.09 ml; carbonic anhydrase (29 kDa), 15.08 ml; and ATP (551 Da), 18.26 ml. The optimal separation range for this column according to the manufacturer’s brochure is 300 kDa to 1 kDa and the void volume 2000 kDa. The K_AV_ for standards in the separation range was calculated using the following equation:
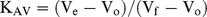
where, V_e_ is the elution volume of the standard; V_o_, is the void volume (7.66 ml); and V_f_, the final volume (18.26 ml). The calibration curve was obtained by representing K_AV_ against the log of the molecular mass for each standard.

### 7. High-sensitivity Isothermal Titration Calorimetry (ITC)

ITC experiments were performed with α2 and β subunits and the oxidized forms of the coenzymes NADPH and NADH using a high precision VP-ITC titration calorimeter (Microcal LLC, Northampton, MA). The purified β subunit (10 and 20 µM) was titrated with NADP^+^ (155 µM) or NAD^+^ (300 µM). Additionally, the α2 subunit (4–13 µM) was titrated with the β subunit (60–190 µM) in the absence or presence of NADP^+^ (300 µM). All measurements were performed in 20 mM Hepes/Na pH 7.5 containing 10 mM MgSO_4_, 100 mM KCl and 2 mM DTT. The binding enthalpy (ΔH), the association constant (*K*_a_) and the stoichiometry (n) were determined simultaneously, considering a model for a single class of binding sites, and the data used to calculate the Gibbs energy (ΔG), and the entropy (ΔS) of binding using well known equations [22]–[24]:

ΔG = −RT ln *K*_a_

ΔG = ΔH−TΔS

### 8. Mass Spectrometry

Purified proteins (10 nmol) were extensively dialyzed against 75 mM ammonium acetate and lyophilized. Aliquots of these samples were subjected to mass spectrometry (MALDI-TOF) using an Applied Biosystems Voyager System 6214 at the facilities of the Instituto de Química-Física Rocasolano (CSIC). In addition, 20 µg of purified refolded-α2 were loaded in a SDS-PAGE gel, stained with Coomassie blue R250 (BioRad), the bands of interest excised and digested with sequencing grade trypsin (Promega) as described by Shevchenko et al. with minor modifications [25]. Disulfide bonds were reduced with 10 mM DTT for 1 h at 57°C and thiol groups alkylated with 55 mM iodoacetamide for 1 h at RT. Peptides were desalted using ZipTip C18 tips (Millipore) and peptide mass fingerprint conducted as previously described [26], using an Autoflex™ mass spectrometer (Bruker Daltonics) in a positive ion reflector mode employing 2,5-dihydroxybenzoic acid as a matrix and an Anchor-Chip™ surface target (Bruker Daltonics). Peak identification and monoisotopic peptide mass assignation were performed automatically using Flex Analysis™ software v. 2.2 (Bruker Daltonics). Database searches were performed against the NCBI non-redundant protein sequence database (http://www.ncbi.nih.gov) using MASCOT (http://matrixscience.com) [27]. The selected search parameters were as follows: tolerance of two missed cleavages; carbamidomethylation (Cys) and oxidation (Met) as fixed and variable modifications, respectively; and setting peptide tolerance to 100 ppm after close-external calibration. A significant MASCOT probability score (p<0.05) was considered as condition for successful protein identification. Additionally, the protein digest was resuspended in 0.1% (v/v) formic acid and analyzed by RP-LC-MS/MS in an Easy-nLC II system coupled to an ion trap LTQ-Orbitrap-Velos-Pro mass spectrometer (Thermo Fisher Scientific). The peptides were concentrated on-line by reverse phase chromatography using a Bio-Basic C18 RP precolumn (0.1×20 mm; Thermo Fisher Scientific), and then separated using a Bio-Basic C18 RP column (0.075×100 mm; Thermo Fisher Scientific) operating at 0.3 µl/min. The column was equilibrated in 0.1% (v/v) formic acid in water (solvent A) and the peptides were eluted using a 1 hour gradient from 5 to 40% solvent B (0.1% formic acid, 80% acetonitrile). ESI ionization was done using a Nano-bore emitters Stainless Steel ID 30 µm interface (Proxeon). The Orbitrap resolution was set at 30000. Peptides were detected in survey scans from 400 to 1600 amu (1 µscans) followed by 10 data dependent MS/MS scans, using an isolation width of 2 u (in mass-to-charge ratio units), normalized collision energy of 35% and dynamic exclusion applied during 30 s. Peptide identification from raw data was carried out using the SEQUEST algorithm (proteome Discoverer 1.3; Thermo Fisher Scientific). Search was performed against a homemade database containing MAT α2 protein. The following constraints were used: tryptic cleavage after Arg and Lys, up to two missed cleavage sites; and tolerances of 10 ppm for precursor ions and 0.8 Da for MS/MS fragment ions; optional Met oxidation and Cys carbamidomethylation were allowed. Protein mass fingerprint was carried out at the Protein Chemistry facility of the Centro de Biología Molecular Severo Ochoa (CSIC), a member of the ProteoRed network.

### 9. Gel Electrophoresis and Densitometric Scanning

Samples (30 µl) of different purification steps were prepared for electrophoresis using Laemmli buffer including 10% (v/v) 2-mercaptoethanol and loaded into 10% SDS-PAGE gels. The electrophoresis standards (BioRad) were: phosphorylase B (97400), bovine serum albumin (66200), ovalbumin (45000), carbonic anhydrase (31000) and soybean trypsin inhibitor (21500). In addition, BlueStar Prestained Protein Markers (Nippon Genetics) were also loaded in a separate lane; those include proteins of the following sizes: 180, 130, 100, 75, 63, 48, 35, 28, 17 and 10 kDa. Molecular mass calculations were carried out using Rf data for both types of standards to obtain the corresponding regression line. A representative example is given by logMr = (−0.883×Rf)+5.171. Staining was performed using Coomassie blue R250 (BioRad). Densitometric scanning of the bands was carried out using the ImageJ software 1.37v (http://rsb.info.nih.gov/ij/).

### 10. Protein Concentration Determinations

The protein concentration of the samples was measured routinely using the BioRad protein assay kit (BioRad) and bovine serum albumin as the standard.

### 11. Sequence Analysis

Alignment of β subunit splicing forms versus V1 or members of the RED family was carried out with Blastp (http://blast.ncbi.nlm.nih.gov/). Theoretical molecular mass calculations were carried out using the protein sequences and the Expasy tools (http://web.expasy.org/compute_pi/).

### 12. Analytical Ultracentrifugation

Sedimentation equilibrium experiments were performed in a Beckman Optima XL-A ultracentrifuge using a Ti50 rotor and six channel centerpieces of Epon-charcoal (optical pathlength 12 mm). Samples of purified α2, β and MAT II (0.2–0.5 mg/ml) were centrifuged at 8000, 10000 and 14000 rpm at 293 K. Radial scans at 280 nm were taken at 12, 14 and 16 hours. The three scans were identical, thus indicating that equilibrium conditions were reached. The weight-average molecular mass (M_w_) of the proteins was determined using the program EQASSOC with the partial specific volume of α2, β and MAT II set to 0.7387, 0.7339 and 0.7366 at 293 K as calculated from their amino acid composition.

### 13. N-terminal Amino Acid Sequencing

Proteins (∼20 µg) resolved in SDS-PAGE gels were transferred to Hybond-P membranes (GE Healthcare), stained with Ponceau S staining and the bands of interest excised as previously described [19]. Sequencing was performed at the Proteomic Service of the Centro de Investigaciones Biológicas (CSIC).

### 14. Protein-Protein Docking

Association of MAT II subunits was investigated using Clus-Pro (http://nrc.bu.edu/cluster), a fully automated web-based program for computational docking of protein structures [28]. For this purpose, the structures of α2 (2P02) and β in the presence of NADP^+^ (2YDX) were submitted. The program evaluates billions of putative complexes, retaining a preset number with favorable surface complementarities. A filtering method is then applied for selection of structures with good electrostatic and desolvation free energies, ranking them according to their clustering properties. The models from the balanced option were selected from the ClusPro output, since there was no prior information as to the chemical nature of the interaction, and the top ten proposed were analyzed. Finally, one was selected on the basis of scores and agreement with biochemical data. The model was submitted to crude dynamics and structure regularization using PHENIX [29] and analyzed with PROCHECK [30].

### 15. Statistics

The Student’s t-test for unpaired samples was applied for statistical analysis of the kinetic data using GraphPad Prism v. 5.0 (GraphPad Software, San Diego). Differences were considered significant when p≤0.05.

## Results and Discussion

### Characterization of Recombinant Catalytic α2 Subunits

The ORF encoded by the human *MAT2A* gene was amplified and cloned into different plasmids for heterologous overexpression. Sequencing of the amplicons from several PCR reactions of different RNA samples consistently included two mutations in the ORF, A233V and G193S, as compared to previously reported cDNAs [31]. The catalytic α2 subunits were produced in reduced amounts as soluble His-tagged proteins and in larger quantities as non-tagged proteins in inclusion bodies, from which they were refolded and purified (Fig. 1). Despite the high degree of identity among α1 and α2 amino acid sequences (85%) the protocol developed for refolding of α1 subunits had to be slightly modified [19]. These modifications refer to an increase in the solubilization time and the first refolding step. Better yields were obtained when the dilution at this step was made to 1 M denaturant, and hence to a lower protein concentration. Purified refolded-α2 appeared as a double band on SDS-PAGE gels, their calculated size being 48 and 45.9 kDa according to the mobility of the standards (Fig. 1A). This ∼2 kDa difference was previously reported for α2 subunits either recombinant or purified from lymphocytic leukemia cells and ascribed to the presence of unknown post-translational modifications [2], [12]. The calculated size for the bands of lymphocytic origin was 53 (α2) and 51 kDa (α2′), as well as for the recombinant protein, values that are slightly larger than those obtained in the present study and by theoretical calculations [2], [12]. Purified preparations obtained from other origins such as bovine brain or erythrocytes showed single bands for α2 subunits on SDS-PAGE gels with calculated masses of 48 and 53 kDa, respectively [3], [4]. Recently, mass spectrometry studies identified several post-translational modifications in α2 subunits of mammalian origin, including acetylations [32], ubiquitinations [33] and phosphorylations [34]. However, none of them has been associated with the discrepant mobilities observed.

In order to exclude the possibility of N-terminal proteolysis during α2 refolding and purification procedures, proteins were separated on SDS-PAGE gels, transferred and the bands excised. N-terminal sequencing of both bands confirmed retention of this part of the protein sequence. This result was also confirmed with purified recombinant Met-Ala-(His)_6_-α2, which showed a double band on the stained gels, indicating copurification of both species on His Trap HP columns. Mass spectrometry analysis (MALDI-TOF) of refolded-α2 showed a single peak of 43447 Da that corresponded to the expected mass for the complete subunit. However, a large proportion of the protein precipitated during sample preparation, and thus loss of one of the protein species could take place. Peptide mass fingerprint of α2 and α2′ bands separated on SDS-PAGE gels was then carried out. The same coverage, ∼50% of the MAT α2 sequence, was obtained for both bands (Fig. 2), with no difference that could be ascribed to the presence of a post-translational modification (Table 1). Results for both bands lack three residues of the C-terminal end that may be the result of trypsin digestion or an intrinsic difference between both α2 and α2′ bands. This last option alone cannot explain the apparent 2 kDa difference between SDS-PAGE bands, but could contribute to changes in mobility due to post-translational modifications occurring in the remaining 50% of the sequence that could not be identified in our analysis. Additional sources of anomalous SDS-PAGE mobilities have been reported in several opportunities, and related among others to the presence of structural elements resistant to standard methods of sample preparation or to ion complexes (i.e. neurocalcin) [35]. The data obtained with recombinant α2 and α2′ do not exclude the possibility that in mammalian cells post-translational modifications occur in the peptides identified in the present study, neither that they are the cause for the larger mass values obtained for α2 and α2′ purified from several tissues.

AdoMet synthesis requires production of oligomers with the correct orientation of their subunits, since catalytic sites locate at the interface of the monomers in the dimer with residues of each contributing to them [15], [36]. Therefore, analytical gel filtration chromatography of refolded-α2 was carried out and the protein detected by activity measurements and Dot-Blot (Fig. 3). The active protein exhibited an elution volume of 12.5 ml that corresponded to an 87 kDa oligomer, according to the elution profile of the markers (Fig. 3A and 3D). The calculated size is compatible with a dimeric association state, a result that is in agreement with previous data obtained for recombinant α2 [12]. However, injection of refolded-α2 samples at higher protein concentrations revealed the presence of two peaks, by both activity and Dot-blot, with calculated elution volumes of 11.44 and 12.50 ml, compatible with tetrameric and dimeric forms (Fig. 3B). Analytic ultracentrifugation of the samples confirmed the presence of tetrameric species above 0.2 mg/ml, hence suggesting a concentration dependent equilibrium (data not shown). Such behavior was not previously observed for recombinant α2, but described for refolded-α1 and other proteins such as NAD^+^-dependent dehydrogenases and tryptophan synthase [21], [37]. Production of stable α1 dimers and tetramers was shown to depend on the presence of a disulfide bond between residues 35 and 61 [21], but lack of cysteine at a position equivalent to C61 precludes such mechanism for α2 and many other members of the MAT family [16].

Purified refolded-α2 showed a specific activity for the production of AdoMet of 187.38 nmol/min/mg (Table 2). This value is in agreement with previously published data for the protein purified from lymphocytic cells (200 nmol/min/mg) [2], therefore ruling out a possible effect of the two mutations detected in our amplicons on activity. In contrast, Met-Ala-(His)_6_-α2 showed a ∼5 fold reduction in the specific activity to 35.19 nmol/min/mg, thus suggesting an effect of the N-terminal tag on this parameter. Addition of tags has been shown to affect both activity and expression of α1 subunits [10] and of other MATs [38], therefore given the high degree of identity among MAT catalytic subunits a similar behavior can be expected. The reason for a longer N-terminal to decrease MAT specific activity is not clear, but may rely on subtle changes in folding affecting indirectly the active site. Based on these data the use of refolded-α2 was preferred for further experiments. Kinetics for the substrates, methionine and ATP, were then carried out with the refolded protein, the calculated values appearing in Tables 3 and 4. The S_0.5_ values obtained for methionine were 5-fold higher than those previously reported by De La Rosa et al. for recombinant α2-dimers (80 µM), but no comparison regarding ATP affinity could be done since that study did not include such data [12].

### Characterization of Recombinant Regulatory β Subunits

The ORF encoded by the human *MAT2B* gene (V1 form) was amplified and cloned for heterologous overexpression. Recombinant soluble regulatory β subunits were produced as GST fusion proteins that were excised with TEV protease in the last purification steps, the resultant protein exhibiting a calculated mass of 37.7 kDa on SDS-PAGE gels (Fig. 1B). This value is in agreement with the theoretical calculations of 37552 Da and the 38 kDa bands reported from mammalian cell purifications [2]–[4]. Our purified protein did not show coelution with *E. coli* α subunits as previously described for His-tagged β by LeGros et al. [39]. Gel filtration chromatography profiles of the purified β subunit showed an elution volume of 86 ml that correspond with a calculated 32 kDa protein according to the elution position of the markers and that is compatible with a monomeric association state (Fig. 4). In contrast, analytical ultracentrifugation of the purified β subunit indicated an average particle size of 1.4, as referred to the theoretical molecular mass, hence suggesting a mixture of monomeric and dimeric species. Again, both techniques required the use of different protein concentration ranges, and hence the divergent behavior suggested the presence of the protein in a concentration dependent equilibrium. These results are in concordance with crystallization experiments in which monomeric (2YDY) and dimeric (2YDX) forms of the β subunit were obtained (Fig. 5).

### Production and Characterization of Recombinant MAT II

Incubation of equimolar amounts of purified refolded-α2 and β proteins allowed production of the hetero-oligomers containing catalytic and regulatory subunits (MAT II). Binding of refolded-α2 to β subunits induced a 5-fold reduction of MAT specific activity as compared to α2 dimers (Table 2) and MAT II purified from lymphocytic cells [2]. Increased affinities for both methionine (∼10 fold) and ATP (∼3.5 fold) were also detected as compared with α2 homo-oligomers (Tables 3 and 4), the S_0.5_ value for methionine being similar to that previously obtained for recombinant MAT II, and thus reproducing the effects described for the regulatory subunit in affinity [39]. The S_0.5_ for methionine shown by recombinant hetero-oligomers was ∼2 fold higher than that obtained with purified MAT II of different tissues (3.3–16 µM) [2], [12], although S_0.5_ values for ATP were similar [2], [3]. Thus, recombinant hetero-oligomers reproduced the regulatory effects of the β subunit previously reported.

The hetero-oligomers were further characterized by analytical gel filtration chromatography. Elution occurs as a single peak at a volume (11.13 ml) corresponding to proteins of ∼170 kDa, according to the elution profile of the markers (Fig. 3C and 3D). This behavior is compatible with a tetrameric association state with a 2∶2 catalytic/regulatory subunit ratio. A similar size, 175 and 160 kDa, was calculated for purified MAT II from lymphocytes and bovine brain [2], [3]. This fact, together with the estimated size of the subunits obtained in SDS-PAGE, suggested three putative hetero-oligomeric associations: (α2β)_2_, α2α2′β_2_ and (α2′β)_2_ [2]. In order to further analyze the hetero-oligomeric association we loaded samples of the peak obtained after gel filtration chromatography on SDS-PAGE gels (Fig. 1B). Densitometric scanning of the stained gels showed (α2+α2′)/β subunit ratios of 1.78±0.17, also compatible with a trimeric association of the type α2_2_β, α2′_2_β or α2α2′β. Data of hydrodynamic techniques, such as gel filtration chromatography, depend not only on the size, but also on the shape of the molecules analyzed. Thus, it is possible that the discrepant results observed with the same sample are due to the shape of the hetero-oligomer that favors elution at a smaller volume. Additional characterization of the MAT II oligomer was attempted by analytical ultracentrifugation, but protein aggregation during the process precluded this type of analysis (data not shown). Hence, to further clarify this point binding between α2 and β subunits was studied by ITC (Fig. 6). The interaction between both subunits was characterized by a high binding affinity (*K*_a_ = 2.7×10^8^ M^−1^) as compared to similar data for a variety of oligomers that range from 10^4^–10^16^ M^−1^ [40], [41]. Additionally, the stoichiometry of the process (n = 0.45) suggested production of a heterotrimer with a 2∶1 (α2+α2′) to β subunit ratio (Fig. 6A and Table 5). Therefore, all these results together indicate hetero-trimeric association of MAT II.

### The Role of NADP^+^ binding to the Regulatory β Subunit

While this work was in due course, a crystal structure of the β subunit including NADP^+^ was obtained (2YDX, Fig. 5), but the role of this compound has not been studied. Previous analysis of the β subunit sequence revealed high homology to NADP^+^ binding proteins classified within the PFAM 04321 oxidoreductase family, its structure including a FAD/NADP^+^ Rossman fold predicted to start at residue 29 (Fig. 7) [1]. Therefore, we decided to analyze NADP^+^ binding to the purified β subunit using ITC (Fig. 6B). The results indicated considerable NADP^+^ binding affinity to this subunit (*K*_a_ = 5.8×10^5^ M^−1^) with a calculated dissociation constant of *K*_d_ = 1.7 µM (Table 5) and a 1∶1 stoichiometry (n = 1). These values revealed the existence of a single NADP^+^ binding site per subunit, together with a dissociation constant in the range reported for other proteins binding this cofactor [23], [42]–[44]. Binding of NADP^+^ to the β subunit is entropically driven, being the enthalpic contribution very small and unfavorable (Table 5). Similar measurements carried out with NAD^+^ revealed lack of binding to the protein, even in assays performed at different temperatures, thus indicating the specificity of the binding site for NADP^+^ (Fig. 6B top inset). Analysis of the cofactor’s binding site observed in the reported crystal structure (2YDX) showed that the phosphate of NADP^+^ is tightly coordinated with the β protein (Fig. 8). Moreover, the interaction produced between this phosphate and R62 seems essential, as this residue is making a network of interactions that conform the NADP^+^ binding pocket (Fig. 8A). All these observations are in agreement with the discrimination between NADP^+^ and NAD^+^ cofactors observed in our experiments.

### Effects of NADP^+^ Binding to the Regulatory Subunit on the Hetero-oligomer

Binding of NADP^+^ to the regulatory β subunit may affect MAT II hetero-oligomerization. Thus, in order to analyze this possibility we studied α2 to β subunit binding in the presence (Fig. 6C) or absence of the coenzyme using ITC (Fig. 6A, Table 5). The data indicated that the process was more favorable when NADP^+^ was present (*K*_a_≥4.0×10^8^ M^−1^), hence suggesting cooperativity between the cofactor and the β subunit while binding to α2. Although the calculated 1.5-fold increase in affinity may seem small, the value reported is at the limit for reliable estimation of the binding affinity, and hence the affinity increase due to the presence of NADP^+^ might be larger. Moreover, the influence of the presence of NADP^+^ is more obvious if the enthalpic and entropic contributions are compared; the presence of NADP^+^ makes the binding enthalpy less favorable and the binding entropy less unfavorable (Table 5). Therefore, binding of β and α2 subunits might proceed together with a conformational change that could be favored by previous production of the β:NADP^+^ complex. Analysis of this possibility by comparison of crystal structures of β in the absence or presence of the cofactor showed that three regions of the polypeptide chain became ordered when NADP^+^ is bound (Fig. 5). Those are: F60-A77 (region A), A95-N113 (region B) and D325-F333 (region C); among them only regions A and B interact directly with the cofactor, whereas regions A and C are involved in β to β interactions in the dimer.

The increased binding affinity between catalytic and regulatory subunits shown in the presence of NADP^+^ could also affect MAT II kinetics. Therefore, purified MAT II was incubated in the presence of 50 µM NADP^+^ and kinetics for both substrates, methionine and ATP, performed. No significant changes were induced by the cofactor in V_max_ or the affinities for both substrates (Tables 2, 3, and 4). However, this lack of effect could rely on the preservation of NADP^+^ binding to the β subunit during purification, before hetero-oligomer production. To analyze this option mutants on the putative cofactor’s binding site were prepared, including G38V in the Wierenga’s motif (GXXGXXG) and the double mutant Y159F/K163A of the putative catalytic triad of the RED family of reductases/epimerases/dehydrogenases (Fig. 7 and 8B). Heterologous expression of G38V yielded no soluble protein, thus being excluded from the study. On the other hand, purified Y159F/K163A-β subunits appeared as a band of 37.7 kDa on SDS-PAGE gels (Fig. 1C), in agreement with theoretical calculations [12]. NADP^+^ binding to this double mutant was then analyzed by ITC and a K_d_>150 µM was calculated (Fig. 6B bottom inset). Therefore, these substitutions lead to a dramatic reduction (>50-fold) in the binding affinity as compared to the wild type β subunit (K_d_ = 1.7 µM), hence indicating severe disruption of NADP^+^ binding in the mutant. Association of α2 subunits with Y159F/K163A-β was not precluded and the hetero-oligomers produced are referred as mutant MAT II. Analysis of these hetero-oligomers by analytical gel filtration chromatography showed elution volumes of ∼11.5 ml, corresponding to proteins of ∼165 kDa, according to the elution volume of the standards. Again, densitometric scanning of SDS-PAGE bands of the hetero-oligomers indicated a 2∶1 ratio for (α2+α2′) and mutant β subunits, hence indicating preservation of the trimeric association state previously shown. The effects of mutant β subunits on kinetics were then analyzed, the results indicating a slight increase in V_max_ for the hetero-oligomers as compared to wild type MAT II that are not statistically significant (Table 2). Affinities for the substrates were decreased in mutant MAT II, the effect being significant only for ATP, ∼2-fold (Tables 3 and 4). Addition of NADP^+^ to mutant MAT II did not modify V_max_ or the affinities for the substrates (Tables 2, 3, and 4), as expected for a mutant lacking key residues for cofactor binding.

### Construction of MAT II Structural Models by Docking of α2 and β Subunits

To obtain further insight within the role of NADP^+^ in the association of β to α2 subunits, models of the MAT II hetero-oligomer were prepared using the ClusPro server for protein-protein docking. Noteworthy, all the proposed output models showed the three β subunit regions ordered upon NADP^+^ binding at the hetero-oligomer interface. According to our data, only models satisfying the 2∶1 stoichiometry for α2 and β subunits would have biochemical sense, and hence the one showing the best scores was selected for further analysis (Fig. 9). The proposed model is coherent with the increased affinity for association of both types of subunit in the presence of NADP^+^, since the cofactor seems to stabilize the putative interface regions, A and C. In addition, participation of α2 central domains in the interaction with β would lead to changes in the active site configuration that could explain variations in enzyme kinetics. Such a role of central domains has been previously demonstrated in MAT α1, whose level of oligomerization depends on the stabilization of these domains, hence the differences in substrate affinities shown by MAT I (tetramer) and III (dimer) [21], [36]. Therefore, the model proposed for MAT II would point to a similar mechanism of specific modulation.

### Significance of Different Splicing Forms of the β Subunit in the Role of NADP^+^

Recently, Yang et al. reported the existence of four splicing forms of the regulatory β subunit in hepatoma cells, V1 and V2 showing the highest expression levels [17]. These two splicing forms derive from the use of a different exon 1, hence showing a specific N-terminal sequence, but preserving the Rossman fold, and thus, the ability to bind NADP^+^ (Fig. 10). The other difference relies on their chain length that varies from 334 residues for V1 to 323 amino acids for V2, due to a shorter exon 1 encoding the N-terminal for this last form. Therefore, to obtain insight in the role of the N-terminal in hetero-oligomer assembly we prepared a truncated form of the β subunit on pOPTG-MAT2B. This truncated β contained the conserved sequence between V1 and V2 splicing forms, including the Rossman fold (starting at residue 29). This ΔR29 protein was expressed in *E. coli*, but was prone to aggregation, and hence excluded from the study. Therefore, a second N-terminal deletion was obtained, according to the spontaneous proteolysis detected in initial experiments with a non-tagged recombinant protein. This truncated protein (ΔS16), starting at residue 16 of V1, was purified following the same protocol as for wild type β. The protein exhibited a 34.8 kDa band on SDS-PAGE gels and associated to refolded-α2, leading to the corresponding hetero-oligomer that was named truncated MAT II (Fig. 1D). Analytical gel filtration chromatography of the truncated MAT II showed elution at ∼11.5 ml, compatible with a protein of ∼165 kDa according to the elution volume of the standards. Densitometric scanning of SDS-PAGE bands of the hetero-oligomer again indicated a refolded-α2 to truncated-β ratio of 2∶1, suggesting production of hetero-trimers.

The effect of N-terminal deletion on kinetics was then explored. A significant increase in V_max_ (1.7-fold) was observed, together with decreases in the affinities for the substrates as compared to wild type MAT II (Table 2, 3, and 4). Reductions in affinity were larger for methionine (5-fold) than for ATP (2-fold), indicating a stronger effect on the methionine binding site. These results suggest a role for the N-terminal of the β subunit in the regulatory effects of α2 catalysis that have been described to date [1]. Comparison of kinetic data of MAT II with those of mutated (lacking the NADP^+^ binding site) and truncated hetero-oligomers, showed that changes in V_max_ and affinities for the substrates are larger in the truncated MAT II and intermediate for the mutant MAT II, which showed more variability. Thus, both, elimination of NADP^+^ binding residues and of the N-terminal, modify kinetics of AdoMet synthesis in α2. However, significant effects on V_max_ are only shown in truncated MAT II, thus suggesting that the role of the N-terminal of the regulatory subunit in favoring the conformational changes of the active site leading to catalysis is more important [15], [45]. Unfortunately, the available crystal structures for the β subunit have been obtained from constructs starting at residue 28, hence precluding analysis of the influence that the N-terminal segment may have on the active site.

Truncated β subunits conserve the NADP^+^ binding site and hence the effects of the cofactor on kinetics could be explored. A slight decrease in V_max_ was induced by addition of the cofactor that did not change the affinities for the substrates in truncated MAT II (Tables 2, 3, and 4). Again, these data suggested a role of NADP^+^ binding in the effects exerted by β subunits on the active site, although preservation of NADP^+^ binding during purification of truncated subunits might be the cause of the minimum changes detected. Kinetic results together highlight the role of the N-terminal of V1 in catalysis and show the importance of both the N-terminal and NADP^+^ binding in the reported changes induced by the β subunit in MAT II [2], [12]. Therefore, it can be deduced that V1, V2 and V2a splicing forms that contain the NADP^+^ binding site will be susceptible of regulation by the cofactor, whereas such a regulation will not occur in V2b forms lacking this site (Fig. 10) [6]. Additionally, the expression of β splicing forms with different N-terminals may provide another level of control of α2 catalysis, a fact that could acquire additional relevance for tumor cells that are known to depend on methionine for growth [46].

In summary, our results support the trimeric association state of the MAT II hetero-oligomer. ITC data indicate that formation of the α2_2_β complex is enthalpically driven and characterized by high affinity. Moreover, production of the β/NADP^+^ complex is characterized by moderate affinity and the α2_2_β oligomer is further strengthened when NADP^+^ is bound to the regulatory β subunit. Therefore, production of looser hetero-oligomers may be the cause of the intermediate kinetic effects observed in a mutant with a severe disruption of cofactor binding. Finally, the N-terminal end of β subunits is important for their regulatory role in catalysis, acting especially on the affinities for the substrates. Thus, differences in this region between splicing forms would provide an additional regulatory mechanism for MAT II.

## References

[1] Pajares MA, Markham GD (2011) Methionine adenosyltransferase (S-adenosylmethionine synthetase). Adv Enzymol Relat Areas Mol Biol 78: 449–452. 22220481 10.1002/9781118105771.ch11

[2] Kotb M, Kredich NM (1985) S-Adenosylmethionine synthetase from human lymphocytes. Purification and characterization. J Biol Chem 260: 3923–3930. 3980460

[3] Mitsui K, Teraoka H, Tsukada K (1988) Complete purification and immunochemical analysis of S-adenosylmethionine synthetase from bovine brain. J Biol Chem 263: 11211–11216. 3403522

[4] Langkamp-Henken B, Geller AM, LeGros HL Jr, Price JO, De la Rosa J, et al (1994) Characterization of distinct forms of methionine adenosyltransferase in nucleated, and mature human erythrocytes and erythroleukemic cells. Biochim Biophys Acta 1201: 397–404. 7803470 10.1016/0304-4165(94)90068-x

[5] Mato JM, Alvarez L, Ortiz P, Pajares MA (1997) S-adenosylmethionine synthesis: molecular mechanisms and clinical implications. Pharmacol Ther 73: 265–280. 9175157 10.1016/s0163-7258(96)00197-0

[6] Yang H, Sadda MR, Yu V, Zeng Y, Lee TD, et al (2003) Induction of human methionine adenosyltransferase 2A expression by tumor necrosis factor alpha. Role of NF-kappa B and AP-1. J Biol Chem 278: 50887–50896. 14530285 10.1074/jbc.M307600200

[7] Lu SC, Alvarez L, Huang ZZ, Chen L, An W, et al (2001) Methionine adenosyltransferase 1A knockout mice are predisposed to liver injury and exhibit increased expression of genes involved in proliferation. Proc Natl Acad Sci 98: 5560–5565. 11320206 10.1073/pnas.091016398 PMC33252

[8] Martinez-Chantar ML, Vazquez-Chantada M, Ariz U, Martinez N, Varela M, et al (2008) Loss of the glycine N-methyltransferase gene leads to steatosis and hepatocellular carcinoma in mice. Hepatology 47: 1191–1199. 18318442 10.1002/hep.22159 PMC2405897

[9] Katoh Y, Ikura T, Hoshikawa Y, Tashiro S, Ito T, et al (2011) Methionine Adenosyltransferase II Serves as a Transcriptional Corepressor of Maf Oncoprotein. Mol Cell 41: 554–566. 21362551 10.1016/j.molcel.2011.02.018

[10] Reytor E, Perez-Miguelsanz J, Alvarez L, Perez-Sala D, Pajares MA (2009) Conformational signals in the C-terminal domain of methionine adenosyltransferase I/III determine its nucleocytoplasmic distribution. FASEB J 23: 3347–3360. 19497982 10.1096/fj.09-130187

[11] Gibson BA, Kraus WL (2011) Small molecules, big effects: a role for chromatin-localized metabolite biosynthesis in gene regulation. Mol Cell 41: 497–499. 21362545 10.1016/j.molcel.2011.02.019 PMC3133593

[12] De La Rosa J, Ostrowski J, Hryniewicz MM, Kredich NM, Kotb M, et al (1995) Chromosomal localization and catalytic properties of the recombinant alpha subunit of human lymphocyte methionine adenosyltransferase. J Biol Chem 270: 21860–21868. 7665609 10.1074/jbc.270.37.21860

[13] Martinez-Chantar ML, Garcia-Trevijano ER, Latasa MU, Martin-Duce A, Fortes P, et al (2003) Methionine adenosyltransferase II beta subunit gene expression provides a proliferative advantage in human hepatoma. Gastroenterology 124: 940–948. 12671891 10.1053/gast.2003.50151

[14] Delgado M, Perez-Miguelsanz J, Garrido F, Rodriguez-Tarduchy G, Perez-Sala D, et al (2008) Early effects of copper accumulation on methionine metabolism. Cell Mol Life Sci 65: 2080–2090. 18560753 10.1007/s00018-008-8201-4 PMC11131693

[15] Markham GD, Pajares MA (2009) Structure-function relationships in methionine adenosyltransferases. Cell Mol Life Sci 66: 636–648. 18953685 10.1007/s00018-008-8516-1 PMC2643306

[16] Sanchez-Perez GF, Bautista JM, Pajares MA (2004) Methionine adenosyltransferase as a useful molecular systematics tool revealed by phylogenetic and structural analyses. J Mol Biol 335: 693–706. 14687567 10.1016/j.jmb.2003.11.022

[17] Yang H, Ara AI, Magilnick N, Xia M, Ramani K, et al (2008) Expression pattern, regulation, and functions of methionine adenosyltransferase 2beta splicing variants in hepatoma cells. Gastroenterology 134: 281–291. 18045590 10.1053/j.gastro.2007.10.027 PMC2409110

[18] Pajares MA, Duran C, Corrales F, Pliego MM, Mato JM (1992) Modulation of rat liver S-adenosylmethionine synthetase activity by glutathione. J Biol Chem 267: 17598–17605. 1517209

[19] Lopez-Vara MC, Gasset M, Pajares MA (2000) Refolding and characterization of rat liver methionine adenosyltransferase from Escherichia coli inclusion bodies. Prot Expr Purif 19: 219–226. 10.1006/prep.2000.1235 10873534

[20] Gil B, Pajares MA, Mato JM, Alvarez L (1997) Glucocorticoid regulation of hepatic S-adenosylmethionine synthetase gene expression. Endocrinology 138: 1251–1258. 9048633 10.1210/endo.138.3.4967

[21] Sanchez-Perez GF, Gasset M, Calvete JJ, Pajares MA (2003) Role of an intrasubunit disulfide in the association state of the cytosolic homo-oligomer methionine adenosyltransferase. J Biol Chem 278: 7285–7293. 12496263 10.1074/jbc.M210177200

[22] Wiseman T, Williston S, Brandts JF, Lin LN (1989) Rapid measurement of binding constants and heats of binding using a new titration calorimeter. Anal Biochem 179: 131–137. 2757186 10.1016/0003-2697(89)90213-3

[23] Velazquez-Campoy A, Goni G, Peregrina JR, Medina M (2006) Exact analysis of heterotropic interactions in proteins: Characterization of cooperative ligand binding by isothermal titration calorimetry. Biophys J 91: 1887–1904. 16766617 10.1529/biophysj.106.086561 PMC1544317

[24] Velazquez-Campoy A, Leavitt SA, Freire E (2004) Characterization of protein-protein interactions by isothermal titration calorimetry. Methods Mol Biol 261: 35–54. 15064448 10.1385/1-59259-762-9:035

[25] Shevchenko A, Wilm M, Vorm O, Mann M (1996) Mass spectrometric sequencing of proteins silver-stained polyacrylamide gels. Anal Chem 68: 850–858. 8779443 10.1021/ac950914h

[26] Naranjo V, Villar M, Martin-Hernando MP, Vidal D, Hofle U, et al (2007) Proteomic and transcriptomic analyses of differential stress/inflammatory responses in mandibular lymph nodes and oropharyngeal tonsils of European wild boars naturally infected with Mycobacterium bovis. Proteomics 7: 220–231. 17163576 10.1002/pmic.200600527

[27] Perkins DN, Pappin DJ, Creasy DM, Cottrell JS (1999) Probability-based protein identification by searching sequence databases using mass spectrometry data. Electrophoresis 20: 3551–3567. 10612281 10.1002/(SICI)1522-2683(19991201)20:18<3551::AID-ELPS3551>3.0.CO;2-2

[28] Comeau SR, Gatchell DW, Vajda S, Camacho CJ (2004) ClusPro: a fully automated algorithm for protein-protein docking. Nucleic Acids Res 32: W96–99. 15215358 10.1093/nar/gkh354 PMC441492

[29] Adams PD, Afonine PV, Bunkoczi G, Chen VB, Davis IW, et al (2010) PHENIX: a comprehensive Python-based system for macromolecular structure solution. Acta Cryst 66: 213–221. 10.1107/S0907444909052925 PMC2815670 20124702

[30] Laskowski RA, MacArthur MW, Moss DS, Thornton JM (1993) PROCHECK: a program to check the stereochemical quality of protein structures. J Appl Crystallogr 26: 283–291.

[31] Horikawa S, Tsukada K (1992) Molecular cloning and developmental expression of a human kidney S-adenosylmethionine synthetase. FEBS Lett 312: 37–41. 1426236 10.1016/0014-5793(92)81405-b

[32] Choudhary C, Kumar C, Gnad F, Nielsen ML, Rehman M, et al (2009) Lysine acetylation targets protein complexes and co-regulates major cellular functions. Science 325: 834–840. 19608861 10.1126/science.1175371

[33] Kim W, Bennett EJ, Huttlin EL, Guo A, Li J, et al (2011) Systematic and quantitative assessment of the ubiquitin-modified proteome. Mol Cell 44: 325–340. 21906983 10.1016/j.molcel.2011.08.025 PMC3200427

[34] Moritz A, Li Y, Guo A, Villen J, Wang Y, et al (2010) Akt-RSK-S6 kinase signaling networks activated by oncogenic receptor tyrosine kinases. Sci. Signal 3: ra64. 10.1126/scisignal.2000998 PMC3137639 20736484

[35] Kato M, Watanabe Y, Iino S, Takaoka Y, Kobayashi S, et al (1998) Cloning and expression of a cDNA encoding a new neurocalcin isoform (neurocalcin alpha) from bovine brain. Biochem J 331: 871–876. 9560316 10.1042/bj3310871 PMC1219429

[36] Gonzalez B, Pajares MA, Hermoso JA, Alvarez L, Garrido F, et al (2000) The crystal structure of tetrameric methionine adenosyltransferase from rat liver reveals the methionine-binding site. J Mol Biol 300: 363–375. 10873471 10.1006/jmbi.2000.3858

[37] Gross M, Jaenicke R (1994) Proteins under pressure. The influence of high hydrostatic pressure on structure, function and assembly of proteins and protein complexes. Eur J Biochem 221: 617–630. 8174542 10.1111/j.1432-1033.1994.tb18774.x

[38] Garrido F, Estrela S, Alves C, Sanchez-Perez GF, Sillero A, et al (2011) Refolding and characterization of methionine adenosyltransferase from Euglena gracilis. Prot Expr Purif 79: 128–136. 10.1016/j.pep.2011.05.004 21605677

[39] LeGros HL Jr, Halim AB, Geller AM, Kotb M (2000) Cloning, expression, and functional characterization of the beta regulatory subunit of human methionine adenosyltransferase (MAT II). J Biol Chem 275: 2359–2366. 10644686 10.1074/jbc.275.4.2359

[40] Friedman FK, Beychok S (1979) Probes of subunit assembly and reconstitution pathways in multisubunit proteins. Annu Rev Biochem 48: 217–250. 157713 10.1146/annurev.bi.48.070179.001245

[41] Markham GD, Satishchandran C (1988) Identification of the reactive sulfhydryl groups of S-adenosylmethionine synthetase. J Biol Chem 263: 8666–8670. 3288619

[42] Dommaraju SR, Dogovski C, Czabotar PE, Hor L, Smith BJ, et al (2011) Catalytic mechanism and cofactor preference of dihydrodipicolinate reductase from methicillin-resistant Staphylococcus aureus. Arch Biochem Biophys 512: 167–174. 21704017 10.1016/j.abb.2011.06.006

[43] Sanae R, Kurokawa F, Oda M, Ishijima S, Sagami I (2011) Thermodynamic analysis of interactions between cofactor and neuronal nitric oxide synthase. Biochemistry 50: 1714–1722. 21244098 10.1021/bi101575u

[44] Martinez-Julvez M, Medina M, Velazquez-Campoy A (2009) Binding thermodynamics of ferredoxin:NADP+ reductase: two different protein substrates and one energetics. Biophys J 96: 4966–4975. 19527656 10.1016/j.bpj.2009.02.061 PMC2712046

[45] Gonzalez B, Pajares MA, Hermoso JA, Guillerm D, Guillerm G, et al (2003) Crystal structures of methionine adenosyltransferase complexed with substrates and products reveal the methionine-ATP recognition and give insights into the catalytic mechanism. J Mol Biol 331: 407–416. 12888348 10.1016/s0022-2836(03)00728-9

[46] Halpern BC, Clark BR, Hardy DN, Halpern RM, Smith RA (1974) The effect of replacement of methionine by homocystine on survival of malignant and normal adult mammalian cells in culture. Proc Natl Acad Sci 71: 1133–1136. 4524624 10.1073/pnas.71.4.1133 PMC388177

